# *LPL* rs264, *PROCR* rs867186 and *PDGF* rs974819 Gene Polymorphisms in Patients with Unstable Angina

**DOI:** 10.3390/jpm14020213

**Published:** 2024-02-16

**Authors:** Damian Malinowski, Krzysztof Safranow, Andrzej Pawlik

**Affiliations:** 1 Department of Pharmacokinetics and Therapeutic Drug Monitoring, Pomeranian Medical University, 70-111 Szczecin, Poland; damian.malinowski@pum.edu.pl; 2 Department of Biochemistry and Medical Chemistry, Pomeranian Medical University, 70-111 Szczecin, Poland; chrissaf@mp.pl; 3 Department of Physiology, Pomeranian Medical University, 70-111 Szczecin, Poland

**Keywords:** LPL, PROCR, PDGF, Polymorphisms, Unstable Angina

## Abstract

Background: Coronary artery disease is caused by changes in the coronary arteries due to the atherosclerotic process and thrombotic changes. A very important role in the development of the atherosclerotic process in the coronary vessels is played by the inflammatory process and the immune response. Due to the important role of lipids and the coagulation process in the atherosclerotic process, research has also focused on genes affecting lipid metabolism and the coagulation system. Lipoprotein lipase (LPL) is an enzyme that metabolises lipids, hydrolysing triglycerides to produce free fatty acids and glycerol. Protein C (PC) is an essential component of coagulation and fibrinolysis. It is activated on the endothelial surface by the membrane-bound thrombin-thrombomodulin complex. Platelet-derived growth factor (PDGF) has a number of important functions in processes related to fibroblast and smooth muscle cell function. Due to their influence on lipid metabolism and coagulation processes, LPL, PROCR (endothelial cell protein C receptor) and PDGF may affect the atherosclerotic process and, thus, the risk of coronary heart disease. The aim of the study was to examine the associations between the *LPL* rs264, *PROCR* rs867186 and *PDGF* rs974819 gene polymorphisms and the risk of unstable angina and selected clinical parameters. Methods: The study included 232 patients with unstable angina and 144 healthy subjects as the control group. Genotyping was performed using real-time PCR. Results: There were no statistically significant differences in the distribution of the polymorphisms tested between the patients with unstable angina and the control subjects. The results showed associations between the *PROCR* rs867186 and *PDGF* rs974819 polymorphisms and some clinical parameters in patients with unstable angina. In patients with the *PDGF* rs974819 CC genotype, there were increased values for cholesterol and LDL serum levels in comparison with patients with the *PDGF* rs974819 CT and TT genotypes. In patients with the *PROCR* rs867186 AA genotype, HDL serum levels were lower than in patients with the GA genotype. Conclusions: The results of our study did not show that the *LPL* rs264, *PROCR* rs867186 and *PDGF* rs974819 gene polymorphisms were significant risk factors for unstable angina in our population. The results of the study suggest that *PDGF* rs974819 and *PROCR* rs867186 may be associated with some parameters of lipid metabolism.

## 1. Introduction

Coronary artery disease is caused by a decrease in the lumen of the coronary artery caused by the atherosclerotic process and thrombotic changes. A very important role in the development of the atherosclerotic process in the coronary vessels is played by the inflammatory process and the immune response. This process involves lipids, especially LDL, which are absorbed by macrophages. The formed foam cells are settled in the vessel wall, contributing to endothelial damage and atherosclerotic plaque development [1]. On this basis, clotting processes are intensified and thrombotic lesions are formed. Previous studies have shown that at the origin of coronary artery disease, in addition to environmental factors such as dyslipidaemia, obesity, smoking, diabetes mellitus (DM) and hypertension, there are also genetic factors. Polymorphisms of genes regulating lipid metabolism and coagulation processes are currently being studied as risk factors for coronary artery disease [2].

Lipoprotein lipase (LPL) is an enzyme that metabolises lipids, hydrolysing triglycerides to produce free fatty acids and glycerol, which are used as energy sources in the heart and other tissues and can also be stored in adipose tissue [3,4]. LPL is also involved in receptor-mediated lipoprotein uptake. It has been shown that LPL could play a role in the development of disorders of lipoprotein metabolism, diabetes, obesity and atherosclerosis [5]. The *LPL* gene is located on chromosome 8p22. A number of polymorphisms have been detected in it that may alter its expression and affect LPL synthesis [6,7]. Previous studies have indicated that the *LPL* rs264 polymorphism is associated with an increased risk of coronary artery disease [8].

Protein C (PC) is an essential component of coagulation and fibrinolysis. It is activated on the endothelial surface by the membrane-bound thrombin-thrombomodulin complex. Once activated, protein C binds to protein S, which is its cofactor, thereby inactivating the pro-coagulation factors VIIIa and Va and inhibiting the clotting process [9]. The endothelial cell protein C receptor (EPCR) plays an important role in protein C activation. This is an N-glycosylated type I membrane protein encoded by the *PROCR* gene (endothelial cell protein C receptor) [10,11]. The *PROCR* gene is localised on chromosome 20q11.2 and consists of four exons. The rs867186 polymorphism in the *PROCR* gene causes a substitution of serine for glycine at codon 219. Studies have shown that this polymorphism affects the expression of EPCR in the endothelium and plasma protein C levels, thereby influencing the coagulation process [12].

Platelet-derived growth factor (PDGF) has a number of important functions in processes related to fibroblast and smooth muscle cell function. PDGF is mainly produced in platelets but is also found in fibroblasts, smooth muscle cells, endothelial cells and macrophages [13]. Current studies suggest that PDGF has multidirectional effects by participating in various pathophysiological processes. PDGF may participate in the development of atherosclerosis by enhancing chemotaxis and exerting mitogenic effects while increasing neovascularisation [14]. Studies have shown that the *PDGF-D* rs974819 polymorphism is associated with an increased risk of coronary artery disease [15].

The aim of the study was to examine the associations between the *LPL* rs264, *PROCR* rs867186 and *PDGF* rs974819 gene polymorphisms and the risk of unstable angina and selected clinical parameters.

## 2. Methods

### 2.1. Study Subjects

For the study’s purposes, 232 patients (aged 62.07 ± 9.68 years) with unstable angina treated in the Department of Cardiology in the years 2017–2018 were enrolled. An unstable angina diagnosis was made on the basis of typical clinical presentation, including angina at rest associated with acute or transient ST segment or T wave changes in an ECG without increases in the markers of myocardial injury (troponin T and myoglobin) and confirmation of significant coronary artery lumen stenosis (>70%) during a coronary angiography. The exclusion criteria were as follows: a final diagnosis of myocardial infarction based on significant increases in the markers of myocardial injury (troponin T and myoglobin), autoimmune diseases and cancer.

For the control group, 144 healthy subjects (aged 67.4 ± 10.6 years) without a history of inflammatory disease or cancer were enrolled into the study. In this group of patients, no significant coronary lumen stenosis was detected on their coronary angiographies, which were performed for diagnoses of unexplained chest pain. The protocol of the study was approved by the relevant local ethics committee (The Bioethics Committee of the Pomeranian Medical University, Szczecin, Poland, KB-0012/46/17), and written informed consent was obtained from all subjects.

### 2.2. Genetic Study

Genomic DNA was extracted from peripheral blood samples, collected in tubes containing EDTA. After material collection, the blood samples were stored at −80 °C until isolation. A silica-membrane DNA extraction method using a Genomic Mini AX Blood 1000 Spin kit (A&A Biotechnology, Gdańsk, Poland) was used according to the manufacturer’s protocol. The DNA was subsequently standardised to equal concentrations of 20 ng/µL, based on spectrophotometric absorbance measurements (260/280 nm) using a DeNovix DS-11 FX+ spectrophotometer/fluorometer (Wilmington, DE, USA).

Genotyping was performed for the following single nucleotide polymorphisms (SNPs): *PDGF* rs974819, *LPL* rs264 and *PROCR* rs867186) using pre-validated allelic discrimination TaqMan real-time PCR assays (C___1953178_30, C__12104296_20 and C__25620145_10, catalogue number: 4351379, Life Technologies, Waltham, MA, USA) and a TaqMan GTXpress Master Mix (Life Technologies, Waltham, MA, USA). All reactions were run in duplicate in final volumes of 12 µL (reaction temperature profile: 95 °C for 20 s, followed by 40 cycles of 95 °C for 1 s and 60 °C for 20 s).

Fluorescence data were obtained using a ViiA7 Real-Time PCR System (Applied Biosystems, Waltham, MA, USA) after a 40-cycle reaction. Genotypes were assigned to individual samples after analysis with TaqMan Genotyper 1.7.1 software (ThermoFisher Scientific, Waltham, MA, USA).

### 2.3. Statistical Analysis

The genotype distributions concordance with Hardy–Weinberg equilibrium (HWE) was assessed using the χ^2^ test. The χ^2^ test and/or Fisher’s exact test were used to compare the distributions of the genotypes and alleles the between groups. The distribution of the quantitative clinical parameters in the study group differed significantly from the normal distribution (determined by a Shapiro–Wilk test), and so they were compared between groups using the non-parametric Mann–Whitney test. Statistical analysis was performed using STATISTICA PL, ver. 13.3 software (StatSoft, Inc., Tulsa, OK, USA). A *p*-value of <0.05 was considered statistically significant. Our study with 232 patients and 144 controls had sufficient statistical power to detect with 80% probability real differences in the allele frequencies between the groups corresponding to an odds ratio (OR) of 0.61 or 1.57 for *PDGF* rs974819, 0.47 or 1.81 for *LPL* rs264 and 0.28 or 2.24 for *PROCR* rs867186. When a Bonferroni correction for multiple comparisons was applied, the corrected level of statistical significance was *p* < 0.0018.

## 3. Results

The distributions of the *LPL* rs264, *PROCR* rs867186 and *PDGF* rs974819 polymorphisms were in HWE and are presented in Table 1. As shown in Table 1, there were no statistically significant differences in the distributions of the polymorphisms tested between the patients with unstable angina and the control subjects for *LPL* rs264 and *PDGF* rs974819, but for *PROCR* rs867186, there were significant differences determined by a chi-square test (*p* = 0.028), though these were not confirmed by a Fisher’s exact test or during allelic analysis, where the differences were not statistically significant.

Additionally, we compared the distributions of the studied polymorphisms between the patients with unstable angina with and without type 2 diabetes as well as with and without hypertension. Increased frequencies of the carriers of the *LPL* rs264 A allele were found among the patients with type 2 diabetes. This difference reached borderline statistical significance (*p* = 0.05). There were no statistically significant differences in the distributions of the *PROCR* rs867186 and *PDGF* rs974819 polymorphisms between the patients with and without type 2 diabetes (Table 2).

There were also no statistically significant differences in the distributions of the studied polymorphisms between the patients with and without hypertension (Table 3).

Next, we analysed the associations between the studied polymorphisms and selected clinical parameters in the patients with unstable angina. In the patients with the *PROCR* rs867186 AA genotype, the HDL serum levels were lower than those in the patients with the AG genotype (Table 4).

In the patients with the *LPL* rs264 AG genotype, the HDL serum levels were lower than those in the patients with the AA genotype (Table 5).

In the patients with the *PDGF* rs974819 CC genotype, there were increased values of cholesterol and LDL serum levels in comparison with the patients with the *PDGF* rs974819 CT and TT genotypes (Table 6).

Since the associations of the three genetic polymorphisms with nine clinical parameters (unstable angina, diabetes, hypertension, age, BMI, total cholesterol, HDL- and LDL-cholesterol, and TG) were analysed, the Bonferroni-corrected significance level was 0.05/(3*9) = 0.0018. Even the strongest association found in our study (that between the *PDGF* rs974819 genotype and serum LDL-cholesterol) did not reach this level of statistical significance corrected for multiple comparisons.

## 4. Discussion

In this study, we examined the distribution of the *LPL* rs264, *PROCR* rs867186 and *PDGF* rs974819 polymorphisms between patients with unstable angina and control subjects. We found statistically significant differences only in the distributions of the *PROCR* rs867186 polymorphism between the studied groups. We also compared the distributions of these polymorphisms between the patients with and without type 2 diabetes, as well as those with and without hypertension. Increased frequencies of the carriers of the *LPL* rs264 A allele were observed among the patients with type 2 diabetes. Additionally, we analysed the associations between the studied polymorphisms and the selected clinical parameters in patients with unstable angina. Patients with the *PDGF* rs974819 CC genotype had elevated serum cholesterol and LDL levels compared to patients with the *PDGF* rs974819 CT and TT genotypes. Patients with the *PROCR* rs867186 AA genotype had lower serum HDL levels than those with the GA genotype. Patients with the *LPL* rs264 AG genotype had lower serum HDL levels than those with the AA genotype. These associations should be treated with caution as they were statistically non-significant after applying the Bonferroni correction.

Coronary artery disease is caused by occlusion of the coronary artery by atherosclerotic and thrombotic lesions. Currently, there is a search for factors that may affect the formation of lesions in the coronary vessels and the development of ischemic heart disease. The inflammatory process taking place in the coronary vessels plays an important role in the development of atherosclerotic lesions [16]. Also involved are lipids and platelets, which form thrombotic lesions. Due to the important roles of lipids and the coagulation process in the atherosclerotic process, research has also been focused on genes affecting lipid metabolism and the coagulation system. In our study, we examined the polymorphism in the *LPL* gene which affects lipid metabolism and those in the *PROCR* and *PDGF* genes which influence the coagulation process.

Lipoprotein lipase is an enzyme that plays a key role in lipid absorption and metabolism [3,4]. It is responsible for the hydrolysis of triglycerides (TG) in chylomicrons and very low-density lipoproteins (VLDL). It also influences lipid exchange between plasma lipoproteins, as well as HDL synthesis. The high expression of LPL has been found in the heart muscle, where this enzyme is responsible for lipid metabolism, as fatty acids are a very important energy component for heart muscle [5]. It has been shown that blocking the activity of LPL in the heart leads to disturbances in lipoprotein and glucose metabolism [17].

The loss of LPL-derived fatty acids in LPL-deficient mice leads to increased glucose metabolism in the heart, cardiac contractile dysfunction and increased cardiac fibrosis [18,19].

To date, a number of studies have examined the association between various *LPL* gene polymorphisms and lipid metabolism parameters, type 2 diabetes and coronary artery disease [7,20]. It has been indicated that the *LPL* gene polymorphism may influence lipid metabolism parameters and may also be a risk factor for type 2 diabetes and coronary artery disease. Our results did not show that *LPL* rs264 is a significant risk factor for the occurrence of unstable angina, but we found a higher frequency of type 2 diabetes in patients with the *LPL* rs264 AA genotype. In addition, patients with the *LPL* rs264 AA genotype had lower serum HDL levels than those with the GA genotype. To date, the *LPL* rs264 polymorphism has not been widely investigated. Matsuoka et al. did not find an association between the *LPL* rs264 polymorphism and the risk of myocardial infarction [21]. However, Osman et al. found an association between the *LPL* rs264 A allele and coronary artery disease in the Arab population [22]. Holmer et al. showed that the *LPL* rs264 polymorphism may be a susceptibility locus for myocardial infarction [23], while Deo et al. found that this polymorphism may influence lipid metabolism parameters in African and European Americans [24].

Protein C (PC) is an important component of the coagulation and fibrinolysis system. Its activation occurs with the involvement of the endothelial cell protein C receptor (EPCR), located mainly on vascular endothelial cells [25]. Recent studies have shown that the endothelial cell protein C receptor plays an important role not only in coagulation and fibrinolysis but also in many important signalling pathways and pathophysiological processes [26]. This receptor has been shown to be involved in many immune reactions, as well as inflammation, apoptosis and cytoprotection [27].

Polymorphisms in the *PROCR* gene were studied in relation to cardiovascular diseases and embolic-thrombotic complications. The rs867186 polymorphism of the *PROCR* gene has been shown to affect plasma protein C levels and, thus, the coagulation process. Fidalgo et al. showed that the *PROCR* rs867186 polymorphism influences the familial risk of venous thrombosis and protein C deficiency in the Portuguese population [28]. Also, in the Turkish and Swedish populations, an association was observed between the rs867186 polymorphism of the *PROCR* gene and venous thrombosis [29,30]. Olson et al. showed that the rs867186 polymorphism of the *PROCR* gene is associated with the incidence of coronary heart disease, ischemic stroke and mortality in the elderly [31]. Previous studies have suggested that the *PROC* rs867186 polymorphism may be a genetic locus for coronary artery disease. Most of the studies performed suggest an association between the polymorphism and coronary artery disease [32,33]. The results of a meta-analysis by Dennis et al. indicated an association of this polymorphism with venous thromboembolism, while no association with myocardial infarction was shown [12]. The results of our study suggest that the *PROCR* rs867186 gene polymorphism is not a significant genetic risk factor for unstable angina in our population, but this polymorphism may affect some lipid metabolism parameters in patients with coronary artery disease.

Platelet-derived growth factor (PDGF) is involved in the regulation of a number of pathophysiological processes by enhancing chemotaxis, exhibiting a mitogenic effect, constricting blood vessels and increasing angiogenesis and the atherosclerotic process [13,14]. Through its mitogenic effects, PDGF enhances the proliferation of fibroblasts and mesenchymal cells, and PDGF may also be involved in cardiac fibrosis and remodelling. Therefore, numerous studies have investigated the role of PDGF in patients with cardiovascular diseases, especially those with cardiovascular insufficiency and coronary artery disease [34]. The role of *PDGF* gene polymorphisms in patients with coronary artery disease has not been widely studied. Zhou et al. showed that the *PDGF* rs974819 polymorphism is a risk factor for coronary artery disease in Chinese men [35]. Similar results were obtained for the Korean population, where this polymorphism was associated with an increased risk of coronary artery disease [15]. In the Japanese population, the *PDGF* rs974819 polymorphism has been shown to be associated with coronary stenosis index in women [36]. Alehagen et al. examined the association between the *PDGF* rs974819 polymorphism and cardiovascular mortality [37], and the authors found that male carriers of the rs974819 A allele exhibited a 2.7-fold increased cardiovascular mortality risk. In our study, there were no statistically significant differences in the distributions of the *PDGF* rs974819 genotypes between the patients with unstable angina and the control subjects, nor were there associations between the rs974819 genotypes and the studied clinical parameters.

It should be noted that our study had a number of limitations. The first major limitation is that the sample size appears to have been relatively small. A larger cohort would increase the statistical power and improve the overall applicability of the results. In addition, the study population was limited to patients from a single department, potentially affecting the applicability of the results more broadly. Although the study highlights associations between gene polymorphisms and clinical parameters, it does not explain causal relationships. It is necessary to conduct further studies to clarify the mechanisms of influence of the studied polymorphisms on specific clinical parameters. Although the study examined associations with clinical parameters, not all of the relevant parameters could be included. This limitation may have hindered a comprehensive understanding of genetic factors affecting unstable angina. The study focused primarily on the polymorphisms of individual genes and did not broadly explore potential interactions between different genes, and thus, it lacked a more detailed understanding of the full genetic background. In addition, relying on patient self-reports about certain clinical parameters could have affected the accuracy and reliability of the data collected.

Ischemic heart disease is a multifactorial disease, with both environmental and genetic factors playing roles in its pathogenesis. The influence of single genes on the development of ischemic disease is small. An important role is played by the interaction of multiple genes that affect the numerous processes involved in its development. Of the greatest importance in the search for genes associated with the risk of developing ischemic heart disease are the multicenter GWAS studies. Identifying the genes associated with an increased risk of ischemic heart disease could enable better prevention and diagnosis of the disease. The case-control study was primarily aimed at encouraging further multicenter GWAS studies.

## 5. Conclusions

The results of our study did not show that the *LPL* rs264, *PROCR* rs867186 and *PDGF* rs974819 gene polymorphisms were significant risk factors for unstable angina in our population. The results of the study suggest that *PDGF* rs974819 and *PROCR* rs867186 may be associated with some parameters of lipid metabolism. In addition, the *LPL* rs264 polymorphism may be associated with a risk of type 2 diabetes. Investigating the role of *LPL* rs264, *PROCR* rs867186 and *PDGF* rs974819 gene polymorphisms in the pathogenesis of unstable angina requires further multicenter studies.

## Figures and Tables

**Table 1:** Distribution of the *PDGF* rs974819, *LPL* rs264 and *PROCR* rs867186 genotypes and alleles in the patients with unstable angina and the controls.

	Control Group (*n* = 144)		Unstable Angina (*n* = 232)		*p*-value ^^^	Compared Genotypes or Alleles	*p*-value ^#^	OR (95% CI)
	*n*	%	*n*	%				
***PDGF* rs974819**								
**Genotype**								
CC	64	44.44%	117	50.43%		TT + CT vs. CC	0.29	0.79 (0.52–1.19)
CT	69	47.92%	101	43.53%	0.501	TT vs. CT + CC	0.53	0.78 (0.34–1.76)
TT	11	7.64%	14	6.04%		TT vs. CC	0.51	0.70 (0.30–1.62)
						CT vs. CC	0.32	0.80 (0.52–1.23)
						TT vs. CT	0.83	0.87 (0.37–2.03)
**Allele**								
C	197	68.40%	335	72.20%				
T	91	31.60%	129	27.80%		T vs. C	0.28	0.83 (0.61–1.15)
***LPL* rs264**								
**Genotype**								
GG	111	77.08%	179	77.16%		AA + GA vs. GG	1.00	1.00 (0.61–1.63)
GA	29	20.14%	46	19.83%	0.989	AA vs. GA + GG	1.00	1.09 (0.31–3.79)
AA	4	2.78%	7	3.01%		AA vs. GG	1.00	1.09 (0.31–3.79)
						GA vs. GG	1.00	0.98 (0.58–1.66)
						AA vs. GA	1.00	1.10 (0.30–4.10)
**Allele**								
G	251	87.15%	404	87.07%				
A	37	12.85%	60	12.93%		A vs. G	1.00	1.01 (0.65–1.56)
***PROCR* rs867186**								
**Genotype**								
AA	129	89.58%	194	83.62%		GG + AG vs. AA	0.13	1.69 (0.89–3.19)
AG	13	9.03%	38	16.38%	0.028	GG vs. AG + AA	0.15	-
GG	2	1.39%	0	0.00%		GG vs. AA	0.16	-
						AG vs. AA	0.06	1.94 (1.00–3.79)
						GG vs. AG	0.08	-
**Allele**								
A	271	94.10%	426	91.81%				
G	17	5.90%	38	8.19%		G vs. A	0.25	1.42 (0.79–2.57)

^^^, χ^2^ test; ^#^, Fisher’s exact test; HWE, control group *p* = 0.248, unstable angina *p* = 0.252 for *PDGF* rs974819; HWE, control group *p* = 0.253, unstable angina *p* = 0.077 for *LPL* rs264; HWE, control group *p* = 0.072, unstable angina *p* = 0.376 for *PROCR* rs867186.

**Table 2:** Distributions of the *PDGF* rs974819, *LPL* rs264 and *PROCR* rs867186 genotypes and alleles in unstable angina patients with and without diabetes mellitus (DM).

	Without Diabetes Mellitus (*n* = 175)		Diabetes Mellitus (*n* = 57)		*p*-value ^^^	Compared Genotypes or Alleles	*p*-value *	OR (95% CI)
	*n*	%	*n*	%				
***PDGF* rs974819**								
**Genotype**								
CC	90	51.43%	27	47.37%		TT + CT vs. CC	0.65	1.18 (0.65–2.14)
CT	76	43.43%	25	43.86%	0.583	TT vs. CT + CC	0.34	1.77 (0.57–5.53)
TT	9	5.14%	5	8.77%		TT vs. CC	0.33	1.85 (0.57–6.00)
						CT vs. CC	0.87	1.10 (0.59–2.05)
						TT vs. CT	0.52	1.69 (0.52–5.51)
**Allele**								
C	256	73.14%	79	69.30%				
T	94	26.86%	35	30.70%		T vs. C	0.47	1.21 (0.76–1.92)
***LPL* rs264**								
**Genotype**								
GG	139	79.43%	40	70.17%		AA + GA vs. GG	0.15	1.64 (0.84–3.23)
GA	33	18.86%	13	22.81%	0.090	AA vs. GA + GG	0.06	4.33 (0.94–19.95)
AA	3	1.71%	4	7.02%		AA vs. GG	0.06	4.63 (1.00–21.56)
						GA vs. GG	0.44	1.37 (0.66–2.85)
						AA vs. GA	0.19	3.39 (0.66–17.25)
**Allele**								
G	311	88.86%	93	81.58%				
A	39	11.14%	21	18.42%		A vs. G	0.05	1.80 (1.01–3.21)
***PROCR* rs867186**								
**Genotype**								
AA	147	84.00%	47	82.46%		GG + AG vs. AA	0.84	1.12 (0.51–2.47)
AG	28	16.00%	10	17.54%	0.784	GG vs. AG + AA	1.00	-
GG	0	0.00%	0	0.00%		GG vs. AA	1.00	-
						AG vs. AA	0.84	1.12 (0.51–2.47)
						GG vs. AG	1.00	-
**Allele**								
A	322	92.00%	104	91.23%				
G	28	8.00%	10	8.77%		G vs. A	0.84	1.11 (0.52–2.35)

^^^, χ^2^ test; *, Fisher’s exact test.

**Table 3:** Distributions of the *PDGF* rs974819, *LPL* rs264 and *PROCR* rs867186 genotypes and alleles in unstable angina patients with and without arterial hypertension (HA).

	Without Arterial Hypertension (*n* = 87)		Arterial Hypertension (*n* = 145)		*p*-value ^^^	Compared Genotypes or Alleles	*p*-value *	OR (95% CI)
	*n*	%	*n*	%				
***PDGF* rs974819**								
**Genotype**								
CC	40	45.98%	77	53.10%		TT + CT vs. CC	0.28	0.74 (0.43–1.25)
CT	39	44.83%	62	42.76%	0.234	TT vs. CT + CC	0.16	0.43 (0.14–1.27)
TT	8	9.19%	6	4.14%		TT vs. CC	0.14	0.39 (0.13–1.20)
						CT vs. CC	0.57	0.83 (0.48–1.44)
						TT vs. CT	0.25	0.47 (0.15–1.46)
**Allele**								
C	119	68.39%	216	74.48%				
T	55	31.61%	74	25.52%		T vs. C	0.17	0.74 (0.49–1.12)
***LPL* rs264**								
**Genotype**								
GG	68	78.16%	111	76.55%		AA + GA vs. GG	0.87	1.10 (0.58–2.07)
GA	17	19.54%	29	20.00%	0.877	AA vs. GA + GG	0.71	1.52 (0.29–8.00)
AA	2	2.30%	5	3.45%		AA vs. GG	0.71	1.53 (0.29–8.12)
						GA vs. GG	1.00	1.05 (0.54–2.04)
						AA vs. GA	1.00	1.47 (0.26–8.40)
**Allele**								
G	153	87.93%	251	86.55%				
A	21	12.07%	39	13.45%		A vs. G	0.78	1.13 (0.64–2.00)
***PROCR* rs867186**								
**Genotype**								
AA	76	87.36%	118	81.38%		GG + AG vs. AA	0.27	1.58 (0.74–3.37)
AG	11	12.64%	27	18.62%	0.234	GG vs. AG + AA	1.00	-
GG	0	0.00%	0	0.00%		GG vs. AA	1.00	-
						AG vs. AA	0.27	1.58 (0.74–3.37)
						GG vs. AG	1.00	-
**Allele**								
A	163	93.68%	263	90.69%				
G	11	6.32%	27	9.31%		G vs. A	0.30	1.52 (0.74–3.15)

^^^, χ^2^ test; *, Fisher’s exact test.

**Table 4:** Associations between the clinical parameters of the patients with unstable angina and the *PROCR* rs867186 genotypes.

Parameters	*PROCR* rs867186 Genotypes				
		AA		AG	AA vs. AG
	*n*	Mean ± SD	*n*	Mean ± SD	*p* ^&^
Age [years]	194	62.06 ± 9.63	38	62.08 ± 10.05	0.979
BMI [kg/m^2^]	194	28.36 ± 4.04	38	28.45 ± 3.52	0.946
CH [mg/dL]	186	230.64 ± 56.57	37	228.43 ± 55.11	0.870
HDL [mg/dL]	154	45.34 ± 8.52	32	42.00 ± 7.35	0.043
LDL [mg/dL]	154	163.62 ± 50.23	32	164.09 ± 52.58	0.944
TG [mg/dL]	185	141.45 ± 76.15	37	131.32 ± 57.00	0.696

^&^, Mann–Whitney U test; BMI, body mass index; CH, total cholesterol in serum; HDL, high-density cholesterol in serum; LDL, low-density cholesterol in serum; TG, triacylglycerols in serum.

**Table 5:** Associations between the clinical parameters of the patients with unstable angina and the *LPL* rs264 genotypes.

Parameters	*LPL* rs264 Genotypes										
		GG		GA		AA	GG vs. GA	AA vs. GA	GG vs. AA	GG + GA vs. AA	AA + GA vs. GG
	*n*	Mean ± SD	*n*	Mean ± SD	*n*	Mean ± SD	*p* ^&^				
Age [years]	179	61.75 ± 9.38	46	62.98 ± 11.01	7	64.00 ± 8.39	0.445	0.793	0.534	0.577	0.365
BMI [kg/m^2^]	179	28.30 ± 3.73	46	28.76 ± 4.83	7	27.57 ± 3.64	0.996	0.674	0.539	0.558	0.837
CH [mg/dL]	172	231.20 ± 56.71	44	231.27 ± 57.17	7	201.29 ± 28.66	0.950	0.198	0.122	0.128	0.648
HDL [mg/dL]	143	44.37 ± 8.47	38	46.90 ± 8.25	5	40.00 ± 3.74	0.116	0.049	0.279	0.193	0.260
LDL [mg/dL]	143	163.56 ± 50.21	38	165.92 ± 54.65	5	151.00 ± 23.48	0.660	0.532	0.585	0.564	0.801
TG [mg/dL]	172	137.99 ± 74.67	43	149.77 ± 69.17	7	121.86 ± 65.22	0.262	0.364	0.468	0.435	0.436

^&^, Mann–Whitney U test; BMI, body mass index; CH, total cholesterol in serum; HDL, high-density cholesterol in serum; LDL, low-density cholesterol in serum; TG, triacylglycerols in serum.

**Table 6:** Associations between the clinical parameters of the patients with unstable angina and the *PDGF* rs974819 genotypes.

Parameters	*PDGF* rs974819 Genotypes										
		CC		CT		TT	CC vs. CT	TT vs. CT	CC vs. TT	CC + CT vs. TT	TT + CT vs. CC
	*n*	Mean ± SD	*n*	Mean ± SD	*n*	Mean ± SD	*p* ^&^				
Age [years]	117	62.00 ± 9.83	101	62.47 ± 9.79	14	59.71 ± 7.56	0.743	0.263	0.375	0.304	0.949
BMI [kg/m^2^]	117	28.61 ± 4.23	101	28.20 ± 3.61	14	27.64 ± 4.11	0.679	0.798	0.677	0.722	0.627
CH [mg/dL]	113	238.42 ± 58.89	96	221.97 ± 53.51	14	221.50 ± 44.60	0.032	0.851	0.384	0.695	0.029
HDL [mg/dL]	93	44.41 ± 8.20	80	45.20 ± 8.63	13	44.69 ± 9.02	0.510	0.618	0.881	0.745	0.585
LDL [mg/dL]	93	174.23 ± 49.50	80	153.58 ± 51.07	13	150.77 ± 40.45	0.006	0.890	0.085	0.305	0.003
TG [mg/dL]	113	142.98 ± 70.73	95	138.07 ± 77.73	14	125.29 ± 64.74	0.341	0.565	0.197	0.320	0.225

^&^, Mann–Whitney U test; BMI, body mass index; CH, total cholesterol in serum; HDL, high-density cholesterol in serum; LDL, low-density cholesterol in serum; TG, triacylglycerols in serum.

## Data Availability

The data presented in this study are available on request from the corresponding author.
